# Experimental Investigation on the Freeze–Thaw Resistance of Steel Fibers Reinforced Rubber Concrete

**DOI:** 10.3390/ma13051260

**Published:** 2020-03-10

**Authors:** Tao Luo, Chi Zhang, Chaowei Sun, Xinchao Zheng, Yanjun Ji, Xiaosa Yuan

**Affiliations:** Shaanxi Key Laboratory of Safety and Durability of Concrete Structures, Xijing University, Xi’an, Shanxi 710123, China; luotao19870426@126.com (T.L.); zc595521884@163.com (C.Z.); zxc1028@foxmail.com (X.Z.); 20170152@xijing.edu.cn (Y.J.); yuanxiaosa2009@163.com (X.Y.)

**Keywords:** steel fiber, rubber concrete, freeze–thaw cycles, compressive strength, four-point bending strength, ICT scanning

## Abstract

The reuse of rubber in concrete results in two major opposing effects: an enhancement in durability and a reduction in mechanical strength. In order to strengthen the mechanical properties of rubber concrete, steel fibers were added in this research. The compressive strength, the four-point bending strength, the mass loss rate, and the relative dynamic elastic modulus of steel fiber reinforced rubber concrete, subjected to cyclic freezing and thawing, were tested. The effects of the content of steel fibers on the freeze–thaw resistance are discussed. The microstructure damage was captured and analyzed by Industrial Computed Tomography (ICT) scanning. Results show that the addition of 2.0% steel fibers can increase the compressive strength of rubber concrete by 26.6% if there is no freeze–thaw effect, but the strengthening effect disappears when subjected to cyclic freeze–thaw. The enhancement of steel fibers on the four-point bending strength is effective under cyclic freeze–thaw. The effect of steel fibers is positive on the mass loss rate but negative on the relative dynamic elastic modulus.

## 1. Introduction

As a result of the growing number of transport vehicles, the number of waste tires keeps increasing. Due to the large amount of ground needed and the threat to the environment caused, burying is not effective for the disposal of waste tires. The reuse of tire rubber as an additive to or replacement of construction materials has become a highly preferable option for energy saving and environmental protection [1,2].

The reuse of rubber in concrete results in two major opposing effects: an enhancement in durability and a reduction in mechanical strength [3,4,5,6,7,8,9,10,11,12,13,14,15,16,17,18,19,20,21,22]. Replacing more than 25% of fine aggregates with rubber crumb caused the compressive strength of concrete to drop significantly [5]. The addition of rubber particles into mortar reduced both the material unit weight and the thermal conductivity [7]. Ultrasonic analysis revealed large reductions in the ultrasonic modulus and high sound absorption for tire-rubber concrete [8]. The reduction in compressive strength at 28 days of age was about 10%–23% for aggregates and 20%–40% for cement replacement. Reduction in modulus of elasticity was 17%–25% in the case of 5%–10% aggregate replacement by chipped rubber and the corresponding reduction for powdered rubber was 18%–36% [10]. The damping coefficient of the rubberized concrete increased by 62% compared with normal concrete [11]. Crumb rubber concrete was found to be effective in absorbing sound [12].

Prior surface treatment of rubber particles can decrease the strength loss caused by introducing crumb rubber into concrete [23]. Mohammandi pointed out that water-soaking rubber treatment resulted in a more uniform distribution of rubber particles in a concrete matrix, less entrapped air in a concrete mixture, and a 22% higher compressive strength for rubberized concrete [24]. Raghavan et al. immersed rubber shreds in NaOH and Ca(OH)_2_ solutions for four months and pointed out that there was a less than 20% change in the stress and strain value [25]. Segre and Joeks surface-treated the rubber particles with NaOH-saturated aqueous solutions for 20 min to increase its adhesion with cement paste [26].

Steel fiber is one of the most popularly used fibers to enhance the mechanical strength of concrete [27,28,29,30,31,32,33,34,35,36,37,38,39,40,41,42,43]. Nguyen et al. indicated that the effects of rubber aggregates and fiber reinforcement could result in better cracking resistance for cement mortar [28]. Jiang et al. pointed out that the constraint effect of steel fiber improved the interfacial bond strength of concrete [29]. Zhang et al. concluded that the incorporation of steel fiber enhanced the freezing-thawing resistance and cracking resistance of concrete containing nano-particles [30]. Zhang et al. pointed out that steel fiber and rubber particles do not improve the frost resistance of roller-compacted concrete in potassium acetate solution [43].

Due to the mechanical strength reduction caused by rubber in concrete, the steel fibers were adopted in this study for enhancing the strength of rubber concrete. The objective of this research is to investigate the freeze–thaw resistance of the steel fiber reinforced rubber concrete and decide the feasibility of using both the advantages of rubber and steel fibers in concrete. The materials and experiments are introduced in Section 2. In Section 3, the effects of steel fiber on the compressive strength, the four-point bending strength, the relative dynamic elastic modulus and the mass loss rate under different freeze–thaw cycles (FTCs) were discussed. The microstructure damage of rubber concrete was captured and analyzed by using Industrial Computed Tomography (ICT) scanning. Some main conclusions were drawn in Section 4.

## 2. Materials and Experiments 

### 2.1. Materials

The details of Portland cement, coarse aggregates, fine aggregates, steel fibers, water reducer, rubber powder and polycarboxylic acid superplasticizer used for preparing the concrete specimens are enumerated below.

Qinling P.O42.5 ordinary Portland cement produced by Shaanxi Yaoxian Cement Co., Ltd. (Tongchuan, China) was used. The physical properties of Portland cement and its chemical compositions are detailed in Table 1 and Table 2, respectively.

The physical properties of the fly ash used in this study are shown in Table 3.

Natural river sand with a fineness module of 2.53 was used as fine aggregates. Natural crushed stones with continuous grading between 5 mm and 20 mm were used as coarse aggregates.

The water reducer produced by Shaanxi Qinfen Building Materials Co., Ltd. (Weinan, China) was used as an admixture, and its physical properties are shown in Table 4. The plasticizer used in the experiment was the polycarboxylic acid superplasticizer (SP) produced by Shandong Yousuo Chemical Technology Co., Ltd. (Linyi, China), and its physical properties are shown in Table 5.

**Figure 1:**
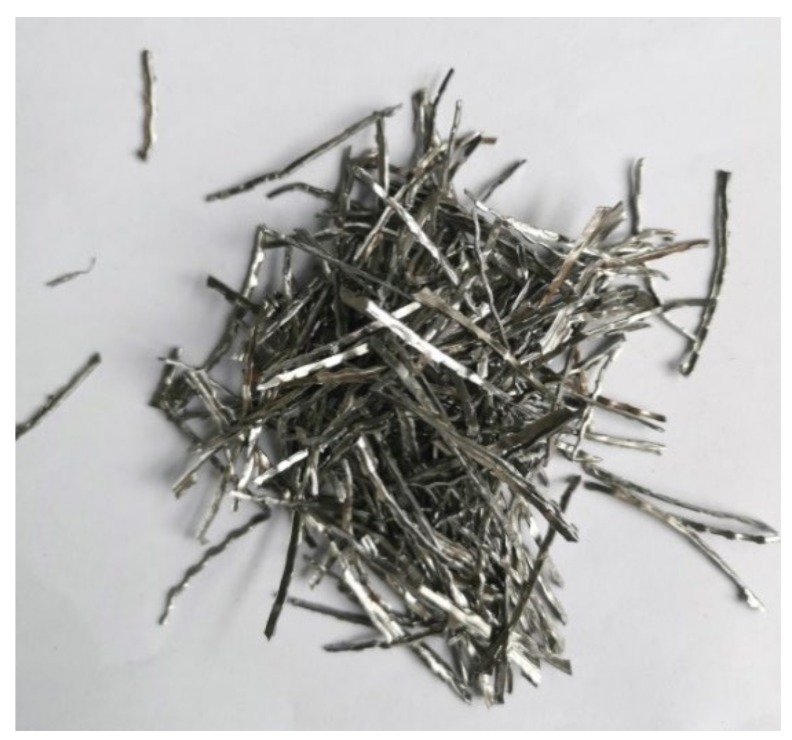
The steel fibers.

The steel fibers, with diameters of 0.5 mm and lengths of 38 mm (Figure 1), were produced by Shuanglian Building Materials Co. Ltd. (Zibo, China). The tensile strength of the steel fiber was 801MPa.

**Figure 2:**
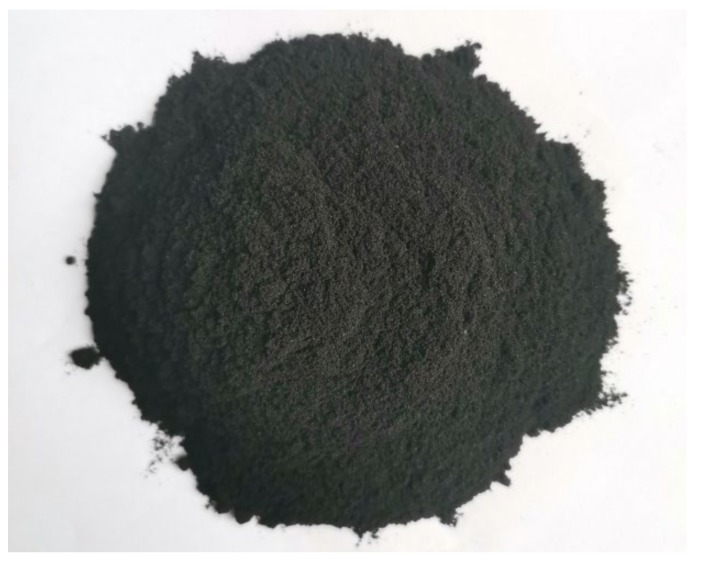
The rubber powder.

The rubber powder with a diameter of 0.125 mm made from used tires (Figure 2) was purchased from Dujiangyan Huayi Rubber Co. Ltd. (Chengdu, China).

### 2.2. Mix Proportions

The mix proportions are listed in Table 6. Four contents of steel fibers (0%, 1%, 1.5% and 2% by volume) were considered.

### 2.3. Experiments

The compressive strength and the four-point bending strength of steel fiber reinforced rubber concrete were considered in this study. The mechanical tests followed the Chinese Standard [44]. A sample with dimensions of 100 mm × 100 mm × 100 mm was used for the compressive strength test. A specimen with dimensions of 100 mm × 100 mm × 400 mm was used for the four-point bending test. All the samples were prepared and cured for 28 days.

**Figure 3:**
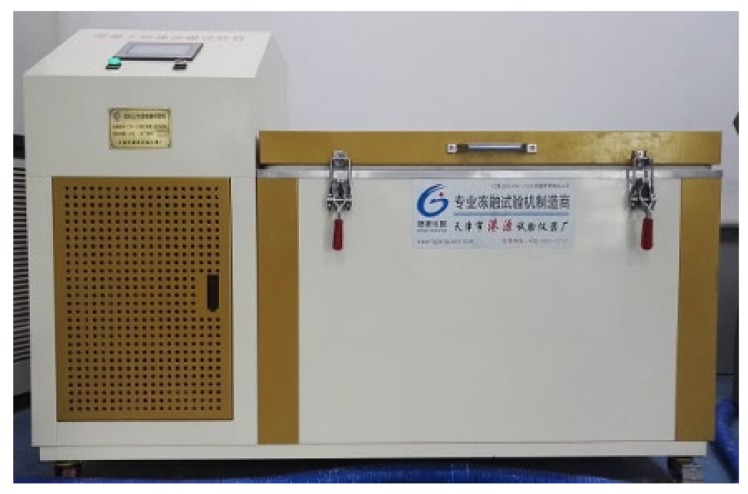
The rapid cyclic freeze–thaw tester.

The rapid cyclic freeze–thaw tester (Figure 3) produced by Tianjin Gangyuan Test Equipment Co., Ltd. (Tianjin, China) was used for the freeze–thaw test. The test procedure followed the Chinese Standard [45]. The temperature range of −20 °C to 20 °C was chosen for the cyclic freeze–thaw test. Each cycle took 4 h.

**Figure 4:**
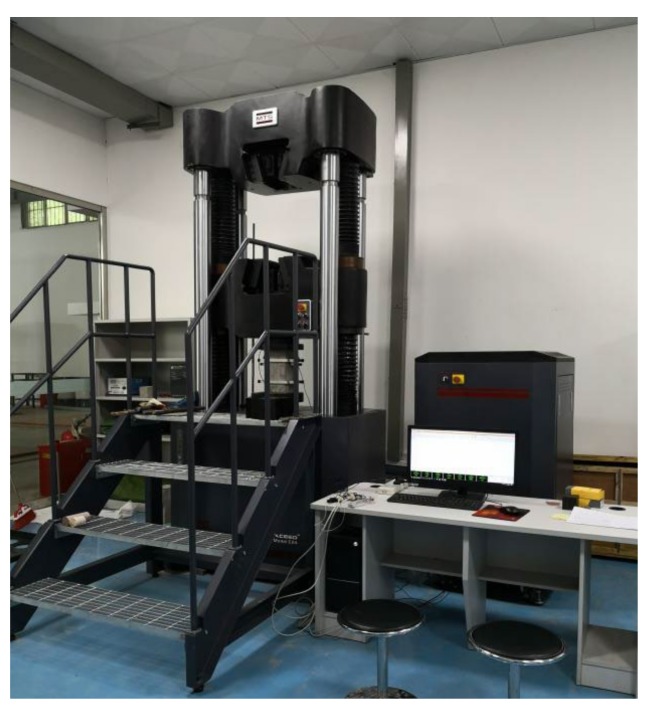
MTS universal testing machine.

**Figure 5:**
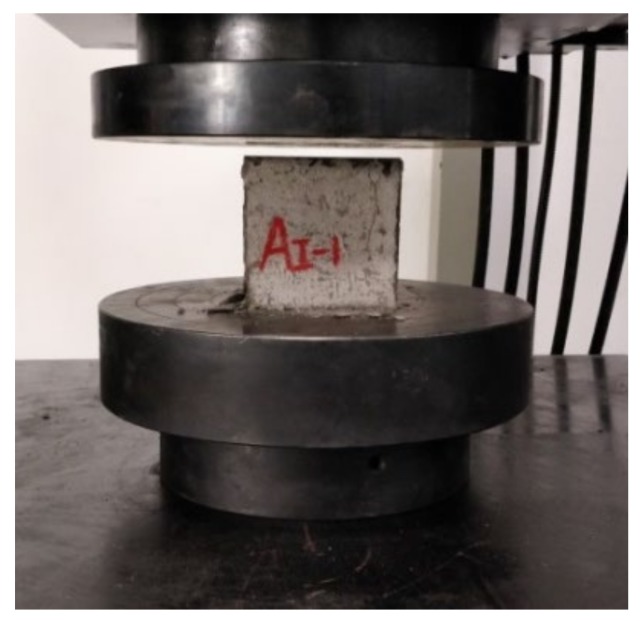
The compressive strength test.

**Figure 6:**
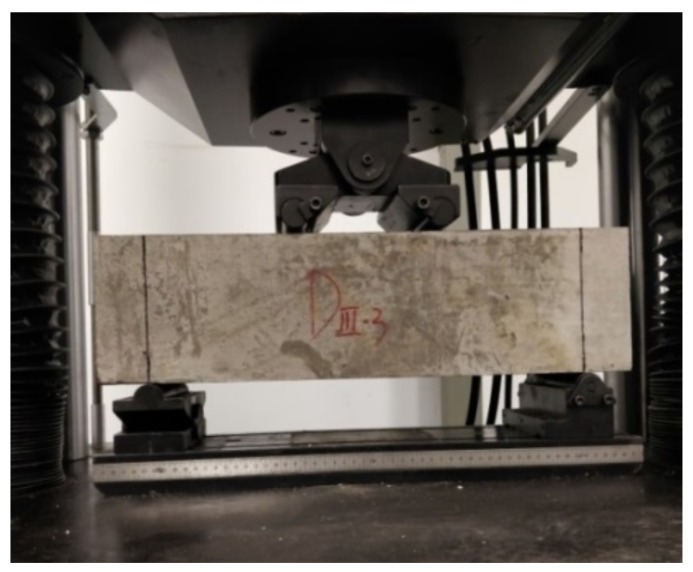
The four-point bending strength test.

Once the freeze–thaw cycles (FTCs) reached 0, 50, 100 and 150 respectively, the samples were tested by the MTS universal testing machine (Shanghai, China) (Figure 4) for compressive strength (Figure 5) and four-point bending strength (Figure 6). Three samples were used for each test. The results were taken from the average of these three samples. 

**Figure 7:**
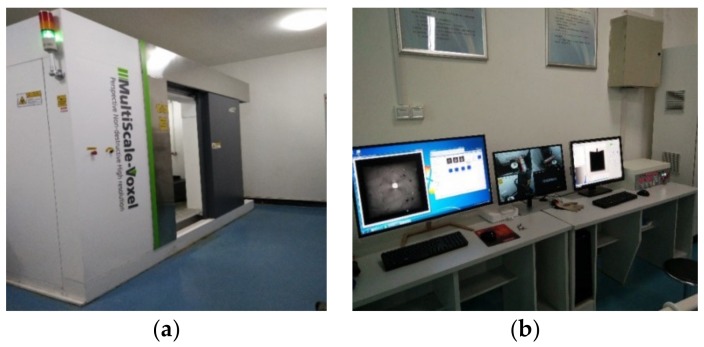
The Industrial Computed Tomography (ICT) equipment. (**a**) The ICT Scanning Equipment; (**b**) The ICT Operating Equipment.

The MS-Voxel 450 Industrial Computed Tomography (ICT) (Tianjin, China) was used for studying the microstructure of the concrete, as shown in Figure 7. The ICT uses a small focus and high-power X-Ray ray source, a high sensitivity detector, and a high precision motion control system, which can ensure high spatial resolution and accurate positioning accuracy in detection. Four samples with four different contents of steel fibers with dimensions of 100 mm × 100 mm × 100 mm were prepared. The samples were scanned by ICT after subjected to the required FTCs. The details of scanning concrete by using ICT are referred to in Liu et al. [46].

## 3. Results and Discussion

The results of the compressive strength, the four-point bending strength, the mass loss rate, the relative dynamic elastic modulus and the microstructure of concrete samples are illustrated and analyzed in the following subsections.

### 3.1. The Compressive Strength

**Figure 8:**
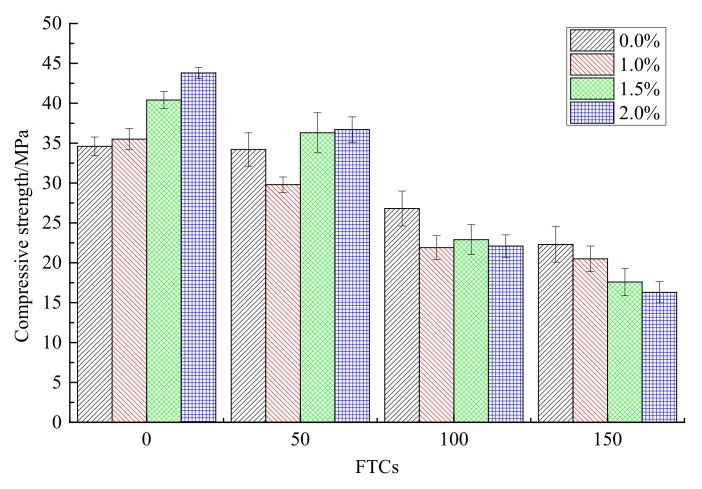
The compressive strength under different freeze–thaw cycles (FTCs).

The compressive strength under different FTCs for different contents of steel fibers is shown in Figure 8. With the increase of FTCs from 0 to 150, the compressive strengths keep decreasing for all contents (0.0%, 1.0%, 1.5% and 2.0%) of steel fibers. In the case of no freeze–thaw cycles, the compressive strength increased with the increase in the contents of steel fibers; this means the addition of steel fibers can enhance the compressive strength of rubber concrete when there is no freeze–thaw effect. For the contents of 2.0% steel fiber added, the compressive strength of rubber concrete improved from 34.6 MPa to 43.8 MPa, which is about a 26.6% increase. With the increasing of FTCs, the strengthening effect of steel fibers on the compressive strength disappears. When the FTCs reached 150, the compressive strength of steel fibers reinforced rubber concrete decreased with the increasing of the contents of steel fibers.

### 3.2. The Four-Point Bending Strength

**Figure 9:**
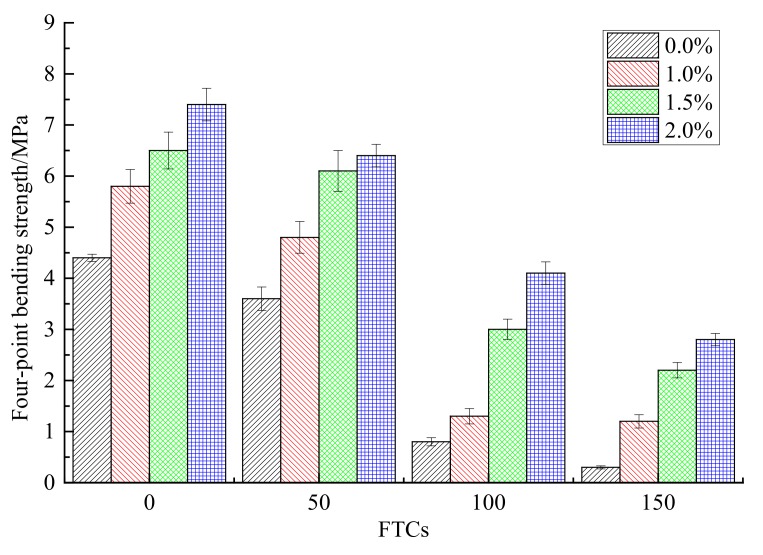
The four-point bending strength under different FTCs.

The four-point bending strength under different FTCs for different contents of steel fibers is shown in Figure 9. With the increase of FTCs from 0 to 150, the four-point bending strength of rubber concrete decreased for all contents (0.0%, 1.0%, 1.5% and 2.0%) of steel fibers added. The four-point bending strength of rubber concrete without steel fibers decreased faster than the rubber concrete reinforced by steel fibers. When the FTCs reached 150, the four-point bending strength of rubber concrete without steel fibers was only about 6.8% reserved, while the concrete reinforced by the content of 2.0% steel fibers reserved about 37.8%. Under each FTC, the four-point bending strength increased with the increase of the contents of steel fibers. For FTCs equal to 0, 50, 100 and 150, the four-point bending strengths of rubber concrete reinforced by the content of 2.0% steel fibers increased by 68.2%, 77.8%, 412.5%, 833.3% respectively, compared to the rubber concrete without steel fibers. The enhancement of steel fibers on the four-point bending strength was much more effective than on the compressive strength. With the increase of FTCs, the strengthening effect on the four-point bending strength increased.

### 3.3. The Mass Loss Rate

**Figure 10:**
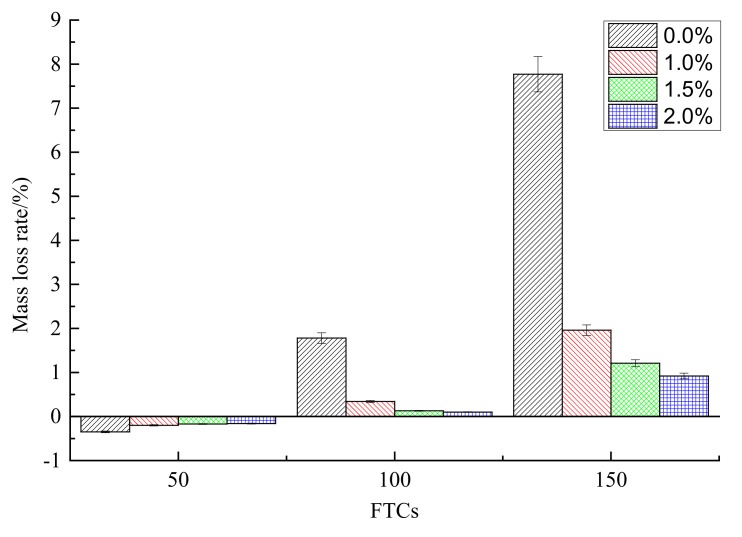
The mass loss rate under different FTCs.

The mass loss rate under different FTCs for different contents of steel fibers is shown in Figure 10. With the increase of FTCs from 50 to 150, the mass loss rates of rubber concrete increased for all contents (0.0%, 1.0%, 1.5% and 2.0%) of steel fibers. The mass loss rate of the rubber concrete without steel fibers increased faster than the rubber concrete reinforced by steel fibers. For each FTC, the mass loss rate of concrete decreased with the increase of the contents of steel fibers. When FTCs reached 150, the mass loss rate of steel fiber reinforced rubber concrete was only 0.92%, which is about 11.8% of the mass loss rate of rubber concrete without steel fibers. Due to the absorption of water, the values of the mass loss rate are negative for FTCs equal to 50. The effect of steel fibers on the mass loss rate of rubber concrete is positive.

### 3.4. The Relative Dynamic Elastic Modulus

**Figure 11:**
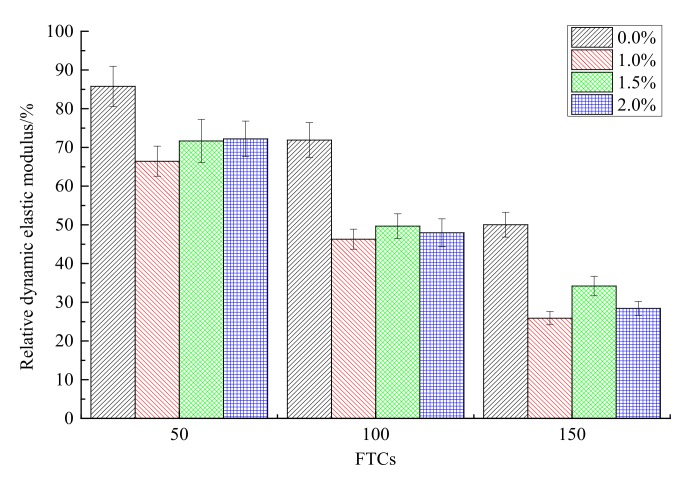
The relative dynamic elastic modulus under different FTCs.

The relative dynamic elastic modulus under different FTCs for different contents of steel fibers is shown in Figure 11. With the increase of FTCs from 50 to 150, the relative dynamic elastic modulus of rubber concrete decreased for all contents (0.0%, 1.0%, 1.5% and 2.0%) of steel fibers. The decreasing speed of the relative dynamic elastic modulus for the concrete without steel fibers added was slower than the concrete reinforced by steel fibers. For example, when the FTCs increased from 50 to 150, the relative dynamic elastic modulus of the concrete without steel fibers decreased by 44.3%, while the relative dynamic elastic modulus of the concrete with 2.0% of steel fibers decreased by 60.6%. For each FTC, the relative dynamic elastic modulus of the rubber concrete reinforced by steel fibers was smaller than the concrete without steel fibers. There is no obvious regulation with the increase of the content of steel fibers. The effect of steel fibers on the relative dynamic elastic modulus is negative.

### 3.5. Microstructure Characterization by ICT

**Figure 12:**
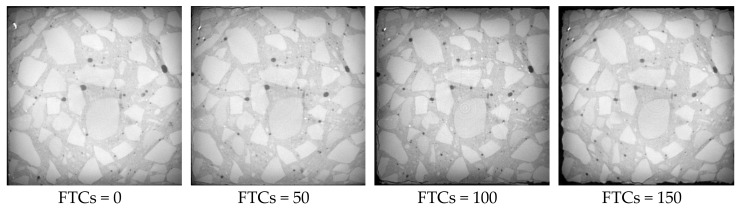
The microstructure of concrete blocks with 0% of steel fibers under different FTCs.

The microstructures of rubber concrete without steel fibers under different FTCs are shown in Figure 12. With the increase of FTCs, the damage caused by cyclic freezing and thawing increased. When FTCs reached 50, there were many cracks appearing along the boundary of the concrete sample. When FTCs reached 100, the external coarse aggregate began to fall off and the boundaries of the specimens became irregular. When FTCs equaled to 150, the damage caused by FTCs gradually invaded the inside of the concrete specimen, the phenomenon of the peeling of the external cement paste and coarse aggregate was more serious, the boundaries of specimen became more irregular, and the expansion of cracks along the interface between aggregate and the cement mortar could be clearly seen. The reason behind this phenomenon can be explained as follows. During the freezing process, the water inside the micro-pores and micro-cracks transforms into ice. The ice pressure causes the expansion of pores and cracks, which induces the damage of concrete. The damage grows gradually from the boundaries of the sample to the inside.

**Figure 13:**
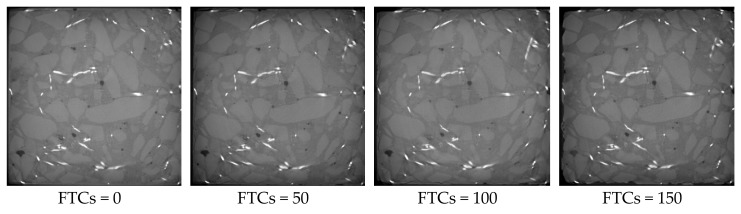
The microstructure of concrete blocks with 1% of steel fibers under different FTCs.

**Figure 14:**
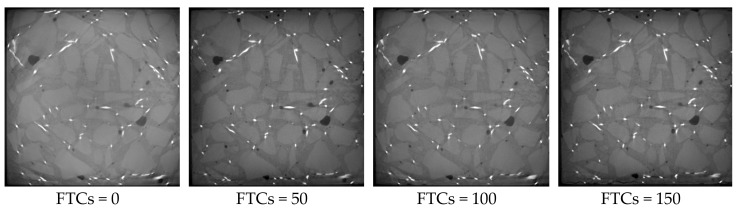
The microstructure of concrete blocks with 1.5% of steel fibers under different FTCs.

**Figure 15:**
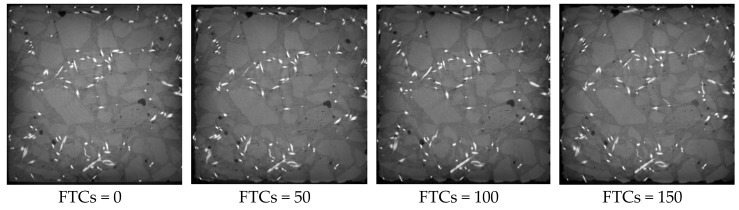
The microstructure of concrete blocks with 2% of steel fibers under different FTCs.

Figure 13, Figure 14 and Figure 15 show the microstructure of rubber concrete reinforced by 1.0%, 1.5% and 2.0% of steel fibers under different FTCs, respectively. The regulation of damage caused by the cyclic freezing and thawing was the same as the concrete without steel fibers. With the increase of FTCs, the cracks grew gradually and resulted in external aggregate falling off and irregular boundaries. Compared to the concrete without steel fibers, the damage mostly happened at the surface where the steel fibers are located, and the damage depth was larger. This is because the bond between steel fiber and the mortar more easily broken by the freezing and thawing cycles than the bond between the aggregate and the mortar. From Figure 13, Figure 14 and Figure 15, the content of steel fibers increased from 1.0% to 2.0%, the phenomenon of the peeling of the external cement paste and coarse aggregate was becoming more serious, and the boundaries of specimens were becoming more irregular.

ImageJ (1.51J8) software was used for doing image analysis for all the sample sections. The outline of the sample section was captured and the area of the section was calculated. The relative areas of the sample sections are shown in Table 7. With the increase in the number of FTCs, the section area decreased. While only one section was taken from one sample, the relative change of different samples cannot reveal the differences caused by different contents of steel fibers.

## 4. Conclusions

The main purpose of this study is to investigate the freeze –thaw resistance of the steel fiber reinforced rubber concrete. Four contents (0.0%, 1.0%, 1.5% and 2.0%) of steel fibers were considered. The rapid cyclic freeze–thaw test, mechanical test and ICT scanning were carried out. The effects of steel fibers on the compressive strength, the four-point bending strength, the relative dynamic elastic modulus and the mass loss rate under different FTCs were discussed. The microstructure damage of rubber concrete was captured and analyzed by using ICT scanning. Based on the results and discussion, some main conclusions can be drawn as follows.

(1) With the increase of FTCs, the compressive strengths of concrete decreased for all contents of steel fibers. The addition of steel fibers can enhance the compressive strength of rubber concrete when there is no freeze–thaw effect. While with the increase of FTCs, the strengthening effect of steel fibers on the compressive strength disappears.
(2) The four-point bending strength of rubber concrete without steel fibers decreases faster than the rubber concrete reinforced by steel fibers. The enhancement of steel fibers on the four-point bending strength is much more effective than on the compressive strength. With the increase of FTCs, the strengthening effect on the four-point bending strength is increasing.
(3) The mass loss rate of the rubber concrete without steel fibers increases faster than the rubber concrete reinforced by steel fibers. The effect of steel fibers on the mass loss rate of rubber concrete is positive.
(4) The decreasing speed of the relative dynamic elastic modulus for the concrete without steel fibers is slower than the concrete reinforced by steel fibers. There is no obvious regulation with the increase of the content of steel fibers. The effect of steel fibers on the relative dynamic elastic modulus is negative.
(5) Compared to the concrete without steel fibers, the damage happens at the surface where steel fibers are located, and the damage depth is larger for the steel fiber reinforced rubber concrete. With the increase of the content of steel fibers, the phenomenon of the peeling of the external cement paste and coarse aggregate is becoming more serious, the boundaries of specimens are becoming more irregular.

## Figures and Tables

**Table 1:** The physical properties of Portland cement.

Density (g/cm^3^)	Fineness (%)	Specific Surface Area (m^2^/g)	Stability	Setting Time (min)		Flexural Strength (MPa)		Compressive Strength (MPa)	
				Initial Setting	Final Setting	3 d	28 d	3 d	28 d
3.10	≤8.0	0.345	Qualified	226	279	5.8	8.6	30.7	50.9

**Table 2:** The chemical compositions of Portland cement (%).

CaO	SiO_2_	Al_2_O_3_	Fe_2_O_3_	MgO	SO_3_	Alkali	Ignition Loss
61.43	22.81	5.62	3.36	1.35	2.17	0.54	2.60

**Table 3:** The physical properties of fly ash.

Fineness (45 µm sieve)	Water Storage Ratio	Burn Loss Ratio	Water Content	SO_3_
**9.6%**	**93%**	**4.8%**	**0.5%**	**1.0%**

**Table 4:** The physical properties of the water reducer.

Water Reduction Ratio (%)	Air-Inducing Ratio (%)	Air Content (%)	Compressive Strength Ratio (%)			
			1 d	3 d	7 d	28 d
14	29	5.0~7.0	220	183	180	165

**Table 5:** The physical properties of polycarboxylic acid superplasticizer (SP).

Appearance	Hydroxyl	PH	Water Content	Solubility
Light yellow to white flakes	22~27	5.0~7.0	≤0.5	Soluble in water and other organic

**Table 6:** The mix proportions.

Mix Group	Composition/kg·m^-3^								
	Cement	Fly Ash	Fine Aggregate	Coarse Aggregate	Water	Water Reducer	Steel Fiber	Rubber Powder	SP
S1	300	18	534	1240	150	0.64	0	10	0.032
S2	300	18	534	1240	150	0.64	78	10	0.032
S3	300	18	534	1240	150	0.64	117	10	0.032
S4	300	18	534	1240	150	0.64	156	10	0.032

**Table 7:** The relative areas of sample sections (%).

Content of Steel Fibers (%)	0 FTCs	50 FTCs	100 FTCs	150 FTCs
0	100	99.68	99.33	98.42
1.0	100	99.71	99.46	99.16
1.5	100	99.82	99.67	99.53
2.0	100	97.89	97.09	97.04
